# Microbial Community Structure Is Most Strongly Associated With Geographical Distance and pH in Salt Lake Sediments

**DOI:** 10.3389/fmicb.2022.920056

**Published:** 2022-06-02

**Authors:** Talitha C. Santini, Lucy Gramenz, Gordon Southam, Carla Zammit

**Affiliations:** ^1^UWA School of Agriculture and Environment, The University of Western Australia, Crawley, WA, Australia; ^2^School of Earth and Environmental Sciences, The University of Queensland, St Lucia, QLD, Australia

**Keywords:** microbial community structure, salt lake, halophiles, iron oxidation, sulfur oxidation

## Abstract

Salt lakes are globally significant microbial habitats, hosting substantial novel microbial diversity and functional capacity. Extremes of salinity and pH both pose major challenges for survival of microbial life in terrestrial and aquatic environments, and are frequently cited as primary influences on microbial diversity across a wide variety of environments. However, few studies have attempted to identify spatial and geochemical contributions to microbial community composition, functional capacity, and environmental tolerances in salt lakes, limiting exploration of novel halophilic and halotolerant microbial species and their potential biotechnological applications. Here, we collected sediment samples from 16 salt lakes at pH values that ranged from pH 4 to 9, distributed across 48,000 km^2^ of the Archaean Yilgarn Craton in southwestern Australia to identify associations between environmental factors and microbial community composition, and used a high throughput culturing approach to identify the limits of salt and pH tolerance during iron and sulfur oxidation in these microbial communities. Geographical distance between lakes was the primary contributor to variation in microbial community composition, with pH identified as the most important geochemical contributor to variation in microbial community composition. Microbial community composition split into two clear groups by pH: *Bacillota* dominated microbial communities in acidic saline lakes, whereas *Euryarchaeota* dominated microbial communities in alkaline saline lakes. Iron oxidation was observed at salinities up to 160 g L^–1^ NaCl at pH values as low as pH 1.5, and sulfur oxidation was observed at salinities up to 160 g L^–1^ NaCl between pH values 2–10, more than doubling previously observed tolerances to NaCl salinity amongst cultivable iron and sulfur oxidizers at these extreme pH values. OTU level diversity in the salt lake microbial communities emerged as the major indicator of iron- and sulfur-oxidizing capacity and environmental tolerances to extremes of pH and salinity. Overall, when bioprospecting for novel microbial functional capacity and environmental tolerances, our study supports sampling from remote, previously unexplored, and maximally distant locations, and prioritizing for OTU level diversity rather than present geochemical conditions.

## Introduction

Salt lakes are globally significant microbial habitats, accounting for around half of Earth’s contemporary lake water (Shiklomanov, 1990). High salinity in salt lakes is often coupled with extremes of pH and high dissolved metals concentrations, imposing strong selection pressures on microbial community composition and function, although the relative importance of various geochemical drivers remains unclear. Understanding the major geochemical and spatial drivers of microbial community composition, functional capacity, and tolerances in salt lakes is of relevance to their utility as early Earth and Martian analogs (Benison et al., 1998; Benison and LaClair, 2003; Benison and Bowen, 2006; Bowen and Benison, 2009; Mormile et al., 2009; Aerts et al., 2019). This will also unlock the potential of salt lakes as repositories of novel microorganisms with functional capacities and tolerances, particularly those relevant to major biotechnological applications like acid saline bioleaching of chalcopyrite (Rea et al., 2015).

Survival at high salinity is predicated on having the functional capacity and cellular machinery to deploy one of two major mechanisms to cope with the osmotic effects of a high salt environment (Oren, 1999, 2011; Watkin and Zammit, 2016; Gunde-Cimerman et al., 2018). These two major mechanisms are: “salt in,” whereby cells counteract the osmotic gradient between the cytoplasm and external environment by accumulating K^+^, and internal cytoplasmic components are designed to function at high salinities; and “salt out,” whereby cells actively reduce the osmotic gradient between the cytoplasm and the external environment by selectively taking up or synthesizing organic compatible solutes in the cytoplasm (including polyols, e.g., glycerol, sugars, e.g., trehalose, and amino acid derivatives, e.g., ectoine) to decrease osmotic pressure (Csonka, 1989; Oren, 1999, 2011). Both strategies incur energetic costs. Survival at high salinities favors those species that can most efficiently compensate for these energetic expenses (Oren, 2011). Salt in strategies are more commonly observed in obligate halophiles (such as the *Halobacteriales*, *Salinibacter*, and *Haloanaerobiales*) as this arrangement maximizes energy yields under saline conditions rather than under low salt conditions; whereas salt out strategies are more commonly observed in halotolerant species (broadly distributed across many microbial groups including the *Bacillota* and *Pseudomonadota*) as their salt tolerance strategies can be up- and down-regulated as required (Oren, 2008). High salt conditions, plus extremes of other environmental variables such as pH, also restrict the range of metabolisms (e.g., acetoclastic/hydrogenotrophic methanogenesis, dissimilatory sulfate reduction, and ammonia and nitrite oxidation) that will yield enough energy (negative Gibbs energy of reaction, −ΔG_*r*_; Amend and Shock, 2001; Shock and Holland, 2007; Oren, 2011). The energetic expense and requisite genetic and cellular machinery required for survival at high salinity plus the limited range of energy-yielding metabolisms under high salt conditions has long been considered to restrict microbial community diversity in salt lakes, playas, and saline lagoons (Oren, 2008), particularly those at extremes of pH. However, recent 16S rRNA amplicon studies have revealed the high microbial diversity of these environments, likely possible in part through tight cycling of substrates and metabolic products via interspecies transfers to counter the energetic expenses of survival (Ley et al., 2006; Johnson et al., 2015) and also in part supported by fluctuating environmental conditions throughout the year (Vavourakis et al., 2016). Recognition of salt lakes as significant repositories of (often novel) microbial diversity (Ley et al., 2006; Mesbah et al., 2007; Demergasso et al., 2008; Narasingarao et al., 2012; Harris et al., 2013; Vavourakis et al., 2016; Aerts et al., 2019; McGonigle et al., 2019; Kajale et al., 2020) strengthens the need to identify and quantify major drivers of microbial community composition, functional capacities, and tolerances within salt lake microbial communities. This knowledge is immediately relevant to identifying hotspots of novel microbial diversity and functional potential, empowering efficient discovery and isolation of new species of biotechnological importance. This has potentially huge value in the mining industry, where salt (and acid) tolerant iron- and sulfur-oxidizing microorganisms are an essential tool to unlock a financially viable pathway for bioleaching of chalcopyrite (CuFeS_2_) (Watling, 2006, 2014). Low grade chalcopyrite now accounts for the majority of the world’s remaining copper reserves (Watling, 2014). Highly halo (acido) philic iron and sulfur oxidizers remain elusive; however, (acidic) salt lakes provide an ideal environment for their evolution (Zammit et al., 2009, 2012), and now, culturing and isolation.

Although some studies have used salt lake environments as targets for culturing of halophiles for particular biotechnological applications (e.g., chalcopyrite bioleaching; Zammit et al., 2009; Rea et al., 2015; Boxall et al., 2017; Khaleque et al., 2018), none have attempted to link microbial community composition and environmental factors with culturable functional capacity such as iron and sulfur oxidation. In attempting to identify drivers of microbial community structure (and less frequently, functional capacity, and environmental tolerances), most previous studies have focussed on one (e.g., Dong et al., 2006; Ley et al., 2006; Jiang et al., 2007; Demergasso et al., 2008; Narasingarao et al., 2012; Montoya et al., 2013; Podell et al., 2014; Tazi et al., 2014), or a limited number (≤7; Rees et al., 2004; Mesbah et al., 2007; Pagaling et al., 2009; Vavourakis et al., 2016; Aerts et al., 2019) of salt lakes or saline lagoons. Only one study of hypersaline lakes (Mora-Ruiz et al., 2018) has evaluated microbial diversity in a larger number of lakes (27 lakes) and connected this with geochemistry, although notably pH was omitted. The limited range in the observed values of geochemical analytes within any one lake or small (≤5) subset of lakes will result in discounting of geochemical properties influencing microbial community composition that may be significant across a broader range of salt lakes, and is likely to miss the role of geographical distance altogether. Indeed, evaluating the roles of both geographical distance and environmental factors in structuring communities in hypersaline lakes was only possible in Mora-Ruiz et al.’s (2018) larger scale study. Those that have attempted to identify geochemical drivers of microbial diversity have variously identified salinity (Jiang et al., 2007; Demergasso et al., 2008; Han et al., 2017; Naghoni et al., 2017; Mora-Ruiz et al., 2018), dissolved ion concentrations (Pagaling et al., 2009; Podell et al., 2014), and redox potential and oxygen availability (Dong et al., 2006; Ley et al., 2006) as the major drivers.

Ecological communities are known to exhibit decreasing compositional similarity as the distance between communities increases (distance-decay; Nekola and White, 1999), attributed to a combination of environmental factors (niche differentiation) and/or dispersal limitation and drift (Nekola and White, 1999; Martiny et al., 2006). Distance effects are likely to become apparent as the distance between sites increases above 10 km (Martiny et al., 2006). The role of spatial distribution in structuring salt lake microbial communities remains largely unstudied despite being identified as a major contributor to variation in microbial community composition in a diverse range of other environments (e.g., rock pools, Langenheder and Ragnarsson, 2007; high altitude freshwater lakes, Reche et al., 2005; plant leaf microbiomes, Finkel et al., 2011; Miura et al., 2017; and grassland soils, Yergeau et al., 2010). Addressing these gaps in our understanding of how spatial and geochemical factors influence microbial community composition, functional capacity, and environmental tolerances requires a comprehensive investigation of salt lake microbial communities over a broad geographical area hosting salt lakes varying widely in their geochemical characteristics.

**FIGURE 1:**
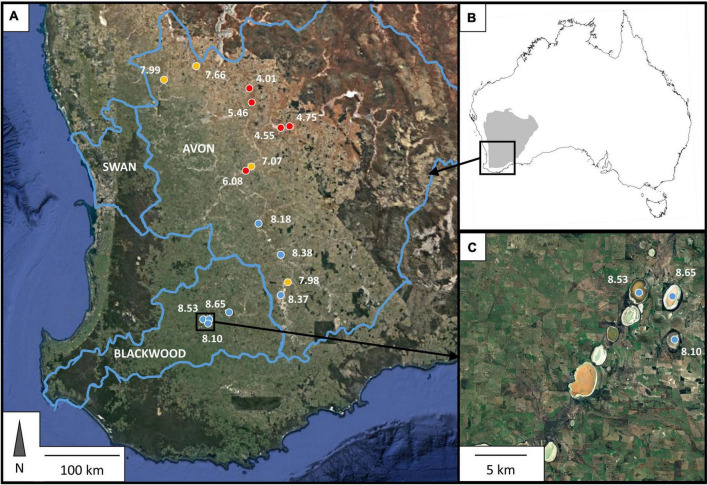
Location of sampling sites within southwest Western Australia, indicated in **(A)** by colored dots, and **(B)** in relation to the continent of Australia (coast outlined in black) and the Yilgarn Craton, which underlies all sites sampled in this study. Three sites were located within 5 km of each other, as shown in panel **(C)**. Blue lines indicate the boundaries of major regional catchments (Geoscience Australia, 1997); names of the catchments are indicated within these boundaries in white capitalized text. Colored dots in **(A,C)** designate different observed pH values: pH < 7—red; 7 ≤ pH ≤ 8—yellow; pH > 8—blue, with exact value of observed pH at the time of sampling shown in white numbers next to each site point.

The Western Australian Yilgarn Craton region provides an ideal field research site in which to investigate associations among microbial community composition, function, and environmental tolerances across salt lakes varying widely in their geochemical properties. The Yilgarn Craton is an ancient (ca. 3,000 Ma old) geological formation underlying the southwest of Western Australia (Figure 1B), and is one of the largest areas (approx. 1.2 million km^2^; deBroekert and Sandiford, 2005) of Archaean crust remaining on Earth. The age of this bedrock combined with the stability of the south-western Australian landscape and lack of export of weathering products has generated a geochemically unique environment characterized by the presence of saline lakes and groundwater bodies at extremes of pH (Bowen and Benison, 2009). In short, uplift of the western and southern margins of the Yilgarn Craton during the early Cenozoic reconfigured regional drainage networks, which previously exported weathering products to the western and southern Australian coasts, such that they became internally draining (Mulcahy et al., 1972; Lillicrap and George, 2010). Subsequent inland accumulation of sediments and salts, coupled with drying of the continental climate as Australia shifted north during the breakup of Gondwana, gave rise to the numerous salt lakes and saline river systems present today (Salama et al., 1992, 1993; Commander et al., 2001; George et al., 2008). Most catchments are internally draining with lakes at the terminus, though some can overflow and connect during exceptional rainfall events (Salama, 1997; Commander et al., 2001). This organization of drainage has been in place for around the past 5 million years (Beard, 1999; Commander et al., 2001), creating a highly geologically stable system in which to investigate associations between microbial community diversity and functional capacity. Despite the relative similarity of underlying geology, climate, and landscape history among the Yilgarn lakes, geochemical properties vary widely (Shand and Degens, 2008; Lillicrap and George, 2010). The exceptional local variability in pH of the saline lakes across the Craton, with pH as low as 2 and as high as 10 (often in proximity to each other), is a key feature of the Yilgarn salt lake systems. Salinity (some over 30%), dissolved metals (Al: up to 0.5 g L^–1^; Fe: up to 0.1 g L^–1^; Cu: up to 1.4 mg L^–1^; Ni: up to 1.9 mg L^–1^), and sulfate (up to 26.5 g L^–1^) and chloride (up to 203 g L^–1^) concentrations are all generally high, although also ranging widely among lakes (Shand and Degens, 2008; Degens and Shand, 2010). The long term geological and climatic stability across the Yilgarn plus the geochemical variation between, and sheer abundance of, salt lakes in this region is a globally unique natural laboratory in which to identify large scale associations among salt lake microbial community composition and functional capacities and tolerances.

The aims of this study were therefore to: (a) quantify the roles of geographical distance and geochemical properties in shaping sediment microbial community composition across the Yilgarn salt lakes; and (b) evaluate pH and salinity tolerances of culturable iron- and sulfur-oxidizing microorganisms, and relate this to salt lake sediment microbial community composition and environmental conditions. Here, we focussed on sediments (and not brines or lake waters), as the major repositories of salt lake microbial diversity (Zaikova et al., 2018). Sediment samples for microbial community compositional analysis, enrichment culturing, and sediment geochemistry were collected from salt lakes distributed across the Yilgarn Craton, removing variation in geology or climatic history as factors driving microbial community composition or functional capacity. Assessment of microbial functional capacity and tolerances focussed on culturable microbial iron and sulfur oxidation capacity at extremes of pH and salinity, given the high biotechnological demand for these functions and tolerances in (chalcopyrite) bioleaching, and the presence of geochemical conditions in the salt lake sediments [extreme pH, high (NaCl) salinity, high metal concentrations] likely to favor selection for these functional capacities and tolerances.

## Materials and Methods

### Sampling Sites and Collection

Sixteen salt lakes distributed over an area of 48,000 km^2^ in southwestern Western Australia were sampled for this study (Figure 1). Lake sediments were collected in early winter 2015 (July). This preceded the onset of heavy winter rains and captured the lakes after only minimal dilution from autumn rains. At each site, thirty subsamples were collected from the shore and littoral zones (using waders to access submerged sediments) with sterile technique and homogenized by combining and mixing all subsamples together in a large sterile Whirlpak polyethylene bag to create one bulk sediment sample for the site. Water depth was generally shallow and did not exceed 1.2 m in any of the lakes at the time of sampling. Samples were stored at 4°C during transport from the field. On arrival at the laboratory, samples were split into three portions: One for geochemical analyses, which was dried to constant weight at 40°C and then stored at room temperature; one for DNA extraction and sequencing, which was stored at –20°C; and one for enrichment culturing, which was stored at 4°C. DNA extraction was completed within 1 month of sample collection and enrichment culturing commenced within 2 weeks of sample collection.

### Geochemical Analyses

pH and EC were determined in 1:5 sediment-water suspensions after shaking end over end for 24 h and settling for 30 min (Rayment and Lyons, 2010). Bicarbonate-extractable P and K (as measures of available P and K) were determined by extraction with a 0.5 M NaHCO_3_ solution at pH 8 (“Colwell P” and “Colwell K”; Colwell, 1965) in a 1:15 ratio with sediment, with P quantified spectrophotometrically by the ammonium molybdate method (Murphy and Riley, 1962; Rayment and Lyons, 2010), using absorbance at 710 nm (Laskov et al., 2007), and K quantified by ICP-OES (Rayment and Lyons, 2010). Exchangeable cations were determined by silver thiourea extract, with a water extraction performed in parallel to account for soluble salts (Rayment and Lyons, 2010) and to provide an estimate of pore water chemistry. Direct collection and analysis of pore water chemistry was not possible at all sites, as some sites were dry at the time of sample collection. Element concentrations in both silver thiourea and water extracts were quantified by ICP-OES. Total, organic, and inorganic C and N were determined by dry combustion furnace with infrared gas detectors for CO_2_ and NO_*x*_ (Rayment and Lyons, 2010), with an acid digest to remove carbonates (Santini et al., 2013).

### DNA Extraction, Sequencing, and Data Analysis

Microbial DNA was extracted from bulk sediment samples (MoBio PowerSoil DNA Isolation kit, MoBio, Carlsbad, CA) and amplified by PCR (Q5 Hot Start High-Fidelity 2X Master Mix Kit, New England Biolabs, Ipswich, MA) using modified versions of universal primers 926F (5′-AAACTYAAAKGAATTGRCGG-3′) and 1392R (5′-ACGGGCGGTGWGTRC-3′), targeting the V6-V8 hypervariable region (Matsuki et al., 2002). PCR was performed in accordance with recommended manufacturer protocols: initial denaturation at 98°C for 30 s, followed by 25 cycles of 98°C for 10 s, 55°C for 30 s, 72°C for 90 s, and a final extension step at 72°C for 2 min. Negative controls (DNAse-free water) were carried in triplicate through DNA extraction and sequencing, and did not pass QA/QC checks in DNA sequencing due to low number of reads returned. DNA sequencing was performed using the Illumina MiSeq platform, and sequence data was processed with QIIME (v 1.8.0) (Caporaso et al., 2010). Multiplex identifiers, primers, chimeras, sequences containing ambiguous base calls, sequences ≤150 bp in length, and sequences containing homo-polymer runs >6 bp were removed from the dataset. An average of 41,228 reads per sample were returned after quality filtering/screening. Operational taxonomic units (OTUs) were defined by clustering at 97% similarity with open reference OTU picking. Sequences were then aligned, phylogenetic trees were created (FastTree; Price et al., 2009) and taxonomy was assigned using BLAST against a curated GreenGenes database (DeSantis et al., 2006). Samples were rarefied to a uniform depth of 15,500 reads per sample. CopyRighter (v 0.46) (Angly et al., 2014) was used to correct the relative abundances of OTUs for variations in 16S rRNA gene copy number.

Alpha diversity was compared between samples using Shannon (H’), Simpson, and Chao1 metrics. Community composition across lake sediments was visualized by distance-based Redundancy Analysis (dbRDA) based on Bray-Curtis dissimilarities, and significant relationships between microbial community composition and geochemical properties of the lake sediments were identified using distance-based multivariate multiple regression (DistLM; Anderson, 2002), implemented in PRIMER, with a forward selection procedure using 9,999 permutations and BIC as selection criterion for model parsimony. Exchangeable Na was removed from the model due to a high proportion of samples (>50%) returning zero values. Water extractable Na was strongly positively correlated with CEC (*r* = 0.97) and water extractable K was strongly positively correlated with Colwell K (*r* = 0.94), and both were removed to avoid multicollinearity. PERMANOVA (Anderson, 2001) and PERMDISP (Anderson et al., 2006), implemented in PRIMER (v 7.0.10, with PERMANOVA + v 1 add-in; PRIMER-E, Plymouth United Kingdom; Clarke, 1993; Clarke and Gorley, 2015), were used to test for statistically significant differences in community composition and dispersion between *post hoc* groups split by pH based on Bray-Curtis dissimilarities, with permutations of residuals under a reduced model using 9,999 permutations. Permutation *P*-values were used unless low unique permutations necessitated the use of Monte Carlo asymptotic *P*-values. Mantel and partial Mantel tests (Mantel, 1967; Legendre and Fortin, 1989) were used to quantify correlations between geographical distance (pair-wise dissimilarities calculated in QIIME using Vincenty’s formula to calculate between-sites distances), microbial community composition (Bray-Curtis dissimilarity matrix), and sediment geochemical factors (Euclidean distance dissimilarity matrix, constructed in GenStat). Key OTUs accounting for the majority of variation between communities were identified using SIMPER, implemented in PRIMER. Sequence data has been submitted to the NCBI Sequence Read Archive under accession number PRJNA563230.

### High Throughput Culturing and Isolation

#### Preparation of Enrichment Culturing Media

Four media were used for enrichment culturing: an iron-oxidizing medium tailored for enrichment of haloacidophiles (Zammit et al., 2012), and three sulfur-oxidizing media [*Sulfolobus* acidophilic medium, Wood et al., 1987; *Thiobacillus neopolitans* neutrophilic medium and *Thiobacillus aquaesulis* alkaliphilic medium (omitting agar), Kelly and Wood, 1998]. The iron-oxidizing medium was only used at acidic pH due to the dominance of abiotic iron oxidation above pH 4 (Ehrlich and Newman, 2009); whereas microbial sulfur oxidation is an important contributor to overall sulfur oxidation rates across a wider pH range (pH 1–10; Baas Becking et al., 1960; Oren, 2011; Sorokin et al., 2011). Three different sulfur-oxidizing media were therefore required to provide appropriate sulfur substrates (e.g., rapid abiotic disproportionation of thiosulfate renders it unsuitable for use at low pH) and nutrients across this wide pH range.

The iron-oxidizing medium contained (g L^–1^): FeSO_4_ (7.6; equivalent to 50 mM Fe^2+^ as substrate), (NH_4_)_2_SO_4_ (3.0), Na_2_SO_4_⋅10H_2_O (3.2), KCl (0.1), K_2_HPO_4_ (0.05), MgSO_4_⋅7H_2_O (0.5), Ca(NO_3_)_2_ (0.01), plus trace elements (mg L^–1^): FeCl_3_⋅6H_2_O (11.0), CuSO_4_⋅5H_2_O (0.5), H_3_BO_3_ (2.0), MnSO_4_⋅H_2_O (2.0), Na_2_MoO_4_⋅2H_2_O (0.8), CoCl_2_⋅6H_2_O (0.6), ZnSO_4_⋅7H_2_O (0.9), and Na_2_SeO_4_ (0.1). pH was adjusted by adding H_2_SO_4_ to achieve pH 1.5, 2, and 2.5. The acidophilic sulfur-oxidizing medium contained (g L^–1^): elemental sulfur (1.3; equivalent to 41 mM S as substrate; sterilized prior to addition), (NH_4_)_2_SO_4_ (0.4), MgSO_4_⋅7H_2_O (0.4), KCl (0.2), K_2_HPO_4_ (0.2), and FeSO_4_⋅7H_2_O (0.01), plus thymol blue as a pH indicator at 0.08 g L^–1^. pH was adjusted by adding H_2_SO_4_ to achieve pH 2 and 3. The neutrophilic sulfur-oxidizing medium contained (g L^–1^): Na_2_S_2_O_3_⋅5H_2_O (5; equivalent to 41 mM S as substrate), NH_4_Cl (0.4), MgSO_4_⋅7H_2_O (0.8), KH_2_PO_4_ (4), K_2_HPO_4_ (4), plus trace elements (mg L^–1^): ZnSO_4_⋅7H_2_O (50), FeSO_4_⋅7H_2_O (50), CaCl_2_ (50), MnCl_2_.6H_2_O (25), CoCl_2_⋅6H_2_O (5), (NH_4_)_2_MoO_4_ (5), and CuSO_4_⋅5H_2_O (2), plus bromothymol blue as a pH indicator at 0.04 g L^–1^. Final pH of this medium was pH 6. The alkaliphilic sulfur-oxidizing medium contained (g L^–1^): Na_2_S_2_O_3_⋅5H_2_O (5; equivalent to 41 mM S as substrate), NH_4_Cl (4), K_2_HPO_4_ (1), MgSO_4_ (1), plus trace elements (mg L^–1^): ZnSO_4_⋅7H_2_O (10), FeSO_4_⋅7H_2_O (10), CaCl_2_ (10), MnCl_2_⋅6H_2_O (5), CoCl_2_⋅6H_2_O (1), (NH_4_)_2_MoO_4_ (1), and CuSO_4_⋅5H_2_O (0.4). pH was adjusted by adding NaOH to achieve pH 8.5 and 10. pH indicators were added as phenol red (saturated solution; 10 mL L^–1^) for the pH 8.5 medium, and thymol blue (salt; 0.08 g L^–1^) for the pH 10 medium.

In all media, salinity was adjusted by adding NaCl to achieve salinities of 20, 60, 100, and 160 g L^–1^. Sodium is the dominant cation and chloride is the dominant anion in Western Australian salt lakes, and both are well correlated with total dissolved solids concentrations (Degens and Shand, 2010). This is consistent with the chemistry of most Australian salt lakes (de Deckker, 1983).

#### High Throughput Enrichment Culturing

A total of 96-well V-bottom polypropylene plates (2 mL capacity per well; Corning CLS3960) were used for high throughput enrichment culturing. Fifty gram of each homogenized bulk sediment sample was mixed with 50 mL sterile ultrapure deionized water and vortexed gently to create a 1:1 sediment-water suspension to enable pipetting. Forty microliter of each sediment suspension was then transferred to triplicate wells in the 96-well plates containing 2 mL of each medium listed above, spanning the full range of pH and salinities listed. Approximately 2 × 10^5^–2 × 10^8^ cells would have been inoculated into each well, based on the quantity of sediment introduced into each well, and reported cell counts in saline lake sediments (10^7^–10^8^ cells g^–1^; Dong et al., 2006; Jiang et al., 2006; Lazar et al., 2011) and soils (10^8^–10^10^ cells g^–1^; Torsvik et al., 1990; Roesch et al., 2007; Raynaud and Nunan, 2014). Sterile control wells containing medium only were used to confirm absence of chemical oxidation of iron and sulfur substrates. Sterile polyethylene film was used to seal the plates, and plates were incubated at 25°C for 21 days on a rotary shaker at 100 rpm. Iron oxidation activity was inferred visually by extent of production of an orange-brown iron oxide precipitate, and scored on a 0–3 rating scale (0—none; 1—minor; 2—moderate; 3—extensive). Sulfur oxidation was inferred by color change of pH indicators in the media, and scored also on a 0–3 rating scale (0—none; 1—minor; 2—moderate; 3—extensive).

## Results and Discussion

### Geographical Distance Underpins Geochemical Properties and Influences Microbial Community Composition Across Western Australian Salt Lakes

Geographical distance between lakes was the primary variable correlated with microbial community composition across Western Australian saline lakes. Differences in geochemistry between lakes were linked to geographical distance, and therefore formed a secondary control on microbial community composition. These factors were distinguished by Mantel and partial Mantel tests. Geographical distance between sites, and lake sediment geochemistry, were both significantly correlated with microbial community composition (Mantel, *p* < 0.05, Table 1). The observed decreases in microbial community similarity as geographical distance between communities increases are expected, in agreement with distance-decay patterns observed globally across microbial, plant, and animal communities (Nekola and White, 1999; Green et al., 2004; Martiny et al., 2006), and hypersaline lakes (Mora-Ruiz et al., 2018). However, geographical distance was also significantly correlated with geochemistry (all variables); and with pH, which was identified as the key geochemical control on microbial community composition by DistLM (Mantel, *p* < 0.01, Table 1). Partial Mantel tests revealed that geographical distance remained significantly correlated with microbial community composition while controlling for either all geochemical variables, or pH only (partial Mantel, *p* < 0.01, Table 1); whereas pH was no longer significantly correlated with microbial community composition once controlling for the effect of geographical distance. All combined geochemical variables remained significantly correlated with microbial community composition while controlling for the effect of geographical distance (partial Mantel, *p* < 0.05, Table 1).

**TABLE 1:** Correlations among microbial community composition (“microbial”), geographical distance (“distance”), and geochemical variables (“all geochem”: All geochemical variables; or “pH”: pH only) as determined by Mantel and partial Mantel tests on dissimilarity matrices.

Comparisons	Mantel r	*P*-value
*Mantel tests*		
Distance × microbial	0.592	**0.001**
All geochem × microbial	0.254	**0.012**
pH × microbial	0.275	**0.011**
Distance × all geochem	0.205	**0.028**
Distance × pH	0.389	**0.003**
*Partial Mantel tests*		
Distance × microbial, controlling for all geochem	0.570	**0.001**
Distance × microbial, controlling for pH	0.547	**0.001**
All geochem × microbial, controlling for distance	0.169	**0.044**
pH × microbial, Controlling for distance	0.061	0.224

*All tests were conducted with 9,999 permutations. Significant correlations (p < 0.05) are indicated with bold P-values.*

**FIGURE 2:**
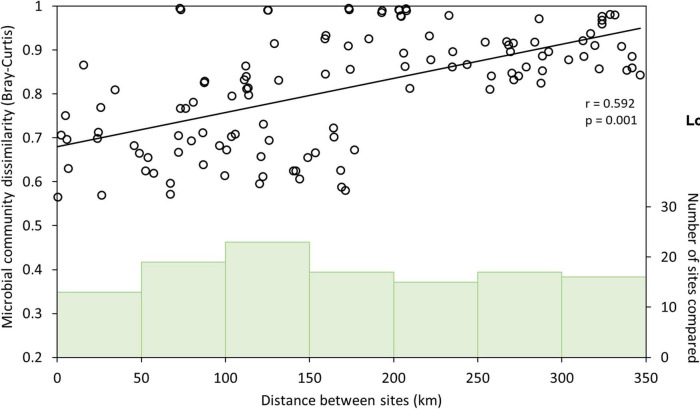
Correlation between microbial community dissimilarity (Bray-Curtis; left *y*-axis) and geographical distance among sampling sites (open circles, *n* = 120). Correlation strength and significance were determined from Mantel tests; line indicates regression line between microbial community dissimilarity and geographical distance. Green vertical bars indicate number of sites compared (right *y*-axis) in each distance category (0–50 km, 50–100 km, 100–150 km, 150–200 km, 200–250 km, 250–300 km, 300–350 km apart) along the *x*-axis.

Inspection of the relationship between geographical distance between sites and microbial community dissimilarity (Figure 2) revealed that sites ≤200 km apart were more variable in their dissimilarity (Bray-Curtis dissimilarities: 0.43–0.99) than those >200 km apart, which were more uniformly and highly dissimilar (Bray-Curtis dissimilarities: 0.81–0.99). The wider variability in the relationship between distance and microbial community dissimilarity at shorter distances implies the influence of factors other than geographical distance on microbial community composition when comparing communities among sites at this scale. Two coherent groups of sites <200 km apart were identified (*n* = 8 sites in each group), and Mantel and partial Mantel tests were used to re-evaluate the effects of geographical distance and geochemical variables on microbial community composition at this smaller spatial scale. Additional comparisons between sites within these groups were not possible as they did not yield complete matrices. Microbial community dissimilarity was significantly correlated with pH in the southern group of sites, whether controlling for the effect of geographical distance or not (Mantel and partial Mantel, *p* < 0.05, Supplementary Table 1); whereas in the northern group of sites, neither geochemistry nor distance had a significant effect on microbial community composition. Geographical distance therefore does not appear to be a significant variable in structuring microbial communities in these salt lakes where they are less than 200 km apart. This is consistent with previous research in a variety of ecosystems summarized in Martiny et al. (2006), showing that environmental factors are important drivers of microbial community composition regardless of scale, but that geographical distance only becomes an important factor in controlling microbial community composition where sites are ≥ 10 km apart. Our study, with up to 346 km distance between sampling sites, enabled the identification of scale-dependent factors in shaping microbial communities in salt lakes, with environmental factors (chiefly pH) dominating over small (< 200 km) spatial scales, and geographical distance becoming increasingly important at greater spatial scales. This extends the work of Mora-Ruiz et al. (2018) by identifying the spatial scales over which geochemistry and geographical distance are significantly correlated with microbial community structure, and explicitly evaluating the interaction between these two properties.

Increasing microbial community dissimilarity as the geographical distance between sites increased (partial Mantel test: *p* < 0.005; Table 1) indicates dispersal limitation between sites and/or drift within sites (Martiny et al., 2006; Nemergut et al., 2013; Xiong et al., 2014). Despite similar geochemical conditions, therefore, individual lakes that are geographically distant may host distinct microbial communities that may or may not include species of interest for biotechnological applications. Interrogation of regional drainage boundaries and catchment and sub-catchment areas shows that the potential for connection between lakes during flood events is minimal (Commander et al., 2001; Supplementary Figure 1), and does not correspond to observed similarities and differences in microbial community composition among lakes. Wind is therefore likely the main mechanism of dispersal among sites, and wind-borne dust dispersal appears to be insufficient to exert a homogenizing effect among sites.

**FIGURE 3:**
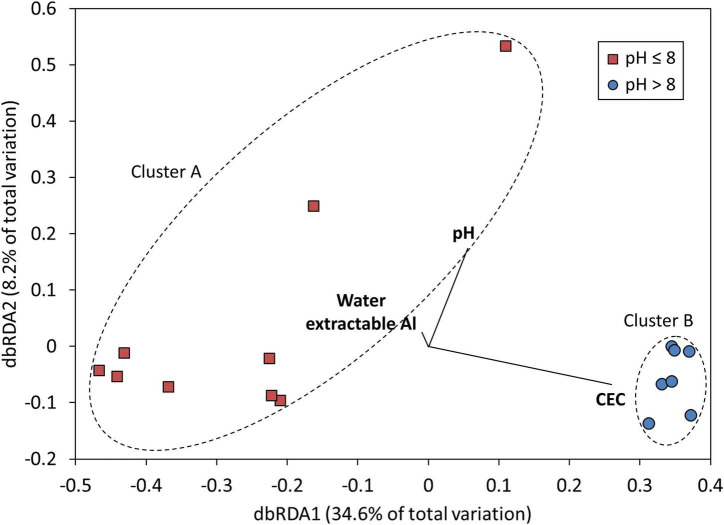
Ordination plot (DistLM results, as visualized by dbRDA) showing relationships between measured geochemical variables and microbial community composition in Western Australian salt lakes. Each point represents microbial community composition in one lake. Lakes are split by sediment pH: Cluster A (PERMANOVA) grouped sites with pH 8 or below (marked with red squares) and Cluster B (PERMANOVA) grouped sites with pH above 8 (marked with blue circles). Dashed lines provide a visual guide to sites grouped within each cluster. Vectors for geochemical variables (in bold) with significant associations with microbial community composition are shown here; variables with non-significant associations were excluded.

Across the entire set of saline lakes sampled in this study, pH was identified as the dominant geochemical control on microbial community composition, accounting for 18% of total variation (DistLM, *p* < 0.01, Table 2 and Figure 3). Marginal tests also identified CEC and water extractable Al as geochemical variables having significant associations with microbial community composition (DistLM, *p* < 0.05, Table 2 and Figure 3), although these became non-significant after accounting for the portion of variation explained by pH and were thus excluded from the BIC model. Models were also created using *R*^2^ and AIC as selection criteria: the AIC model returned the same results as the BIC model, which indicates a parsimonious solution. The *R*^2^ model fitted a greater number of variables (in accordance with expectations of this approach), but overall returned the same results as the AIC and BIC models given that only pH returned a *p*-value of < 0.05 under the sequential tests for the *R*^2^ model (Supplementary Tables 2, 3). The low total amount of variation in microbial community composition explained by geochemical variables (pH only: 18% total) supported the large role of other factors such as geographical distance to variation in microbial community composition across sites. pH has been observed elsewhere to play a key role in structuring microbial communities in freshwater lakes (Xiong et al., 2012) and soils (Fierer and Jackson, 2006; Lauber et al., 2009; Rousk et al., 2009); our results were consistent with these studies. Water extractable Na, Ca, and S were major sources of geochemical (dis) similarity among lakes (Supplementary Figure 2); however, these variables were only weakly linked to variation in microbial community structure (Supplementary Table 2). This emphasizes that sampling regimes focussed only on maximizing geochemical dissimilarities among sampling sites ignores the microbiologically relevant geochemical parameters and will thus result in undersampling of total microbial diversity. Salinity has also been noted as a key driver of microbial community composition in lakes (Yang et al., 2016; Mora-Ruiz et al., 2018) and a range of terrestrial and aquatic environments (Lozupone and Knight, 2007), but was not strongly associated with microbial community composition here. The generally high salinity of all lakes [ranging from 17 to 63 mS cm^–1^ (Supplementary Table 4); approximately 1–3.5% w/v salt, intermediate between the slight (2–5%; Vreeland, 1987) and moderate (3–15%wt; Kushner, 1978) halophile ranges] studied may have been a sufficiently strong and uniform selection pressure as to remove those species incapable of salt tolerance and thus diminish the strength of association between salinity and microbial community structure. Previous studies noting salinity as a key driver (Lozupone and Knight, 2007; Yang et al., 2016; Mora-Ruiz et al., 2018) included a much wider range of salinity values at their study sites, and not all sites sampled in these previous studies were sufficiently saline to require microbial salt tolerance mechanisms for survival.

**TABLE 2:** Results of distance-based multivariate multiple regression (DistLM) based on Bray-Curtis dissimilarities for microbial community structures and measured geochemical variables, using 9,999 permutations under a forward selection procedure with Bayesian Information Criterion as selection criterion.

Environmental characteristic	*P*-value	Percentage of variation explained (%)	Multiple partial correlations with dbRDA axes	
			Axis 1 (34.6% total)	Axis 2 (8.2% total)
pH	0.007	17.98	0.116	0.291
CEC	0.025	14.29	0.534	–0.110
Water extractable Al	0.017	16.05	–0.023	0.054

*P-values and percentage of variation explained are for marginal tests, with only those geochemical variables having a significant P-value (p < 0.05) listed here. Percentages listed with dbRDA axes indicate the percentage of total variation explained by each axis.*

### Bacillota Dominated Microbial Communities in Acidic Saline Lakes; Euryarchaeota Dominated Communities in Alkaline Saline Lakes

**FIGURE 4:**
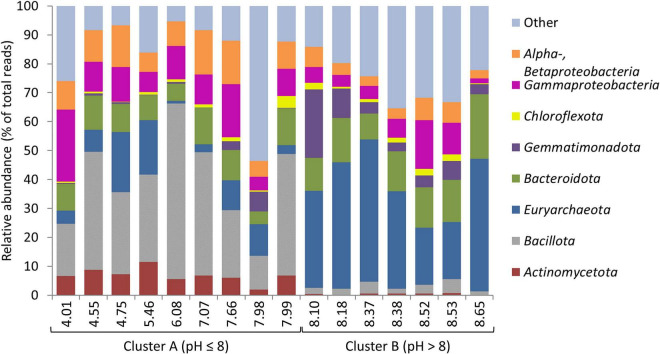
Phylum (and class) level relative abundances within microbial communities from the 16 Western Australian saline lakes sampled in this study. Lakes are arranged in order of ascending pH along the *x*-axis, and grouped into Clusters (A,B) identified by PERMANOVA.

Where most previous studies have been limited to one or two salt lakes, our study, which sampled 16 lakes within a 48,000 km^2^ area, revealed a clear pH control on microbial community composition across salt lakes. Overall, microbial community compositions were consistent with previous studies in hypersaline environments (e.g., Jones et al., 1998; Rees et al., 2004; Oren, 2008; Pagaling et al., 2009; Vavourakis et al., 2016; Han et al., 2017; Mora-Ruiz et al., 2018), dominated by *Pseudomonadota* (*Gammaproteobacteria*), *Bacillota*, and *Euryarchaeota*. Community composition split into two major clusters (PERMANOVA: *p* < 0.001), linked to pH (Figure 3): Cluster A (sediment pH ≤ 8), dominated by *Bacillota*, *Pseudomonadota*, and *Actinomycetota*; and Cluster B (sediment pH > 8), dominated by *Euryarchaeota*, *Bacteroidota*, and *Gemmatimonadota* (Figure 4; SIMPER, Supplementary Table 5 and Supplementary Figure 3). This confirms the significant role of pH in structuring microbial communities across salt lakes as already identified by Mantel tests. OTUs from *Lactobacillus*, *Clostridiaceae*, and *Lachnospiraceae* (all *Bacillota*), *Enterobacteriaceae* (*Pseudomonadota* [*Gammaproteobacteria*]), and *Propionibacteriaceae* (*Actinomycetota*) accounted for 23.64% of relative abundance and 38.79% of similarity across sites within Cluster A (Supplementary Table 5). OTUs from *Halobacteriaceae*, *Natronomonas*, and *Halorhabdus* (all *Euryarchaeota*), *Salinibacter* (*Bacteroidota*), and *Gemmatimonadota* accounted for 35.45% of relative abundance and 38.96% of similarity across sites within Cluster B (Supplementary Table 5). The dominance of *Bacillota*, *Pseudomonadota*, and *Actinomycetota* in acidic to neutral salt lakes and *Euryarchaeota*, *Bacteroidota*, and *Gemmatimonadota* in alkaline salt lakes is broadly consistent with observations from previous studies spanning a range of lake pHs (Pagaling et al., 2009; Casamayor et al., 2013; Han et al., 2017). The dominance of *Halobacteriales* (*Halobacteriaceae*, *Natronomonas*, and *Halorhabdus*) and *Salinibacter* within the alkaline salt lakes, which are lineages known to host obligate halophiles (salt-in strategists), suggests more consistently high salinity conditions in these lakes compared with the acidic salt lakes which tended to host halotolerant, salt-out strategists (Oren, 2008). The two most abundant OTUs within each cluster, *Lactobacillus* sp. (Cluster A) and *Halobacteriaceae* sp. (Cluster B) were the major contributors to dissimilarity between the clusters, accounting for a combined 12.57% of between-cluster dissimilarity (SIMPER; Supplementary Table 5). Although *Lactobacillus* and *Halobacteriaceae* are not known to host iron- and sulfur-oxidizing species, they are well known for their utility in other biotechnological applications such as hydrocarbon degradation (*Halobacteriaceae*; Krzmarzick et al., 2018) and organic acid and polysaccharide production (*Lactobacillus*; Sun et al., 2015). Interrogation of microbial community composition at lower taxonomic ranks revealed putative sulfur oxidizers (*Thiohalorhabdus* spp., *Halothiobacillus* spp.; Kelly and Wood, 2000; Sievert et al., 2000; Sorokin et al., 2008; Whaley-Martin et al., 2019); however, these were present at low relative abundances, as were putative iron oxidizers (*Mariprofundus* spp., *Gallionella* spp.; Hedrich et al., 2011).

### High Tolerance to Acidity and Salinity Among Iron and Sulfur Oxidizers

Enrichment cultures from the saline lakes in this study showed remarkable tolerances to extremes of salinity and pH. Iron oxidation was observed at salinities up to 160 g L^–1^ NaCl at pH values as low as pH 1.5 (Table 3), and sulfur oxidation was observed at salinities up to 160 g L^–1^ NaCl at pH values spanning pH 2–10 (Table 4). Under sulfur- and/or iron-oxidizing enrichment conditions, tolerances for salinity from chloride (up to 70 g L^–1^; Rea et al., 2015) are generally lower than tolerances for other anions (e.g., sulfate: up to 140 g L^–1^; Watling et al., 2010; Rea et al., 2015). The high NaCl concentrations (20–160 g L^–1^) used in this study therefore, pose a particular challenge for iron and sulfur oxidizers, particularly when combined with extremes of pH. Among the iron and sulfur oxidizers, high NaCl tolerances are typically only observed at circumneutral pH [e.g., *Halothiobacillus halophilus* (sulfur oxidizer): tolerance up to 4 M NaCl, pH 6.5–8.4; Kelly and Wood, 2000]. The dominance of NaCl salinity in Western Australian salt lakes sampled here (de Deckker, 1983; Degens and Shand, 2010) has likely favored selection of species capable of tolerating this particular challenge of combined NaCl salinity and extreme pH. Previous studies have reported tolerances of NaCl in pure strains up to 20 g L^–1^ at pH 4 (sulfur oxidation, *A. thiooxidans*; Kamimura et al., 2003, 2005), 60 g L^–1^ at pH 2.5 (iron and sulfur oxidation, *Thiobacillus prosperus*; Huber and Stetter, 1989), and 60 g L^–1^ at pH 1.7–3 (iron oxidation, *A. ferrooxidans*; Nicolle et al., 2009). Higher NaCl tolerances have been observed in enrichment cultures: up to 70 g L^–1^ for sulfur oxidation (pH 3–4.5) and 65 g L^–1^ for iron oxidation (pH 4) (Rea et al., 2015). Our results, more than doubling the known tolerance to NaCl salinity amongst cultivable iron- and sulfur-oxidizing enrichment cultures to 160 g L^–1^ NaCl at acidic pH values (pH 2–4), confirm naturally saline environments as a rich source of novel microbial metabolic capacity. Further work is ongoing to isolate individual species and interrogate metabolic capabilities from metagenome-assembled genomes from these enrichment communities. Future studies should also include considerations of the rates of oxidation processes and biomass growth. Incubations in this study were performed over a limited timeframe, which may fail to capture growth and activity in communities where halophilic iron- and sulfur-oxidizing species are present in low initial relative abundances, or are slow-growing. This study has identified several promising target lakes for future isolation and culturing for biotechnological applications such as bioleaching, and these now require further enrichment studies to focus these isolation efforts. These efforts should focus on Lake Campion, Salt Lake, and Lime Lake for iron oxidation; and Baandee South, Baandee North, Lake De Courcy, Lime Lake, Jilakin, Jobs Lake, Lake Brown, Dumbleyung, and Salt Lake for sulfur oxidation. More detailed temporal assessment of activity during the incubation period of this study would highlight fast responders, which would be the preferred targets for isolation and application in bioleaching as they would be expected to rapidly oxidize substrates in this setting.

**TABLE 3:** Iron oxidation activity in acidic, saline enrichment cultures.

Lake	Sediment pH	pH 1.5				pH 2				pH 2.5			
		Salinity (g L^–1^ NaCl)				Salinity (g L^–1^ NaCl)				Salinity (g L^–1^ NaCl)			
		20	60	100	160	20	60	100	160	20	60	100	160
Jobs Lake	4.01	0	0	0	0	0	0	0	0	0	0	0	0
Lake Campion	4.55	1	1	1	1	1	1	1	1	1	1	1	1
Brown Lake	4.75	0	0	0	0	0	0	0	0	0	0	0	0
Lake Mcdermott	5.46	0	0	0	0	0	0	0	0	0	0	0	0
Baandee south	6.08	0	0	0	0	0	0	0	0	0	0	0	0
Baandee north	7.07	0	0	0	0	0	0	0	0	0	0	0	0
Lake De Courcy	7.66	0	0	0	0	0	1	1	1	1	1	1	1
North Lake Grace	7.98	0	0	0	0	0	0	0	0	2	2	2	2
Lake Lucille	7.99	0	0	0	0	0	0	0	0	1	1	1	1
Lime Lake	8.10	1	0	0	0	2	3	2	3	3	3	3	3
Kurrenkutten	8.18	0	0	0	0	0	0	0	0	2	2	2	2
Lake Grace	8.37	0	0	0	0	1	1	1	1	2	2	2	2
Jilakin	8.38	0	0	0	0	1	1	1	1	3	3	3	3
Dumbleyung	8.52	0	0	0	0	1	1	1	0	2	2	2	2
Parkeyerring	8.53	0	0	0	0	1	0	0	0	3	3	3	3
Salt Lake	8.65	1	1	0	0	3	3	3	3	3	3	3	3

*Activity was assessed via visual observation of extent of orange-brown precipitate (iron oxyhydroxide) formation according to the following scale: 0—none (no shading); 1—minor (light blue); 2—moderate (mid blue); 3—extensive (dark blue).*

**TABLE 4:** Sulfur oxidation activity in saline enrichment cultures.

Lake	Sediment pH	Acidophilic (S°) medium, pH 2				Acidophilic (S°) medium, pH 3				Neutrophilic (thiosulfate) medium, pH 6				Alkaliphilic (thiosulfate) medium, pH 8.5				Alkaliphilic (thiosulfate) medium, pH 10			
		Salinity (g L^–1^ NaCl)				Salinity (g L^–1^ NaCl)				Salinity (g L^–1^ NaCl)				Salinity (g L^–1^ NaCl)				Salinity (g L^–1^ NaCl)			
		20	60	100	160	20	60	100	160	20	60	100	160	20	60	100	160	20	60	100	160
Jobs Lake	4.01	1	0	0	0	2	2	1	0	0	0	0	0	1	1	1	1	3	3	3	1
Lake Campion	4.55	0	0	0	0	2	2	2	1	2	1	1	1	2	1	1	0	3	2	0	0
Brown Lake	4.75	1	0	0	0	1	1	1	0	0	0	0	0	2	1	1	1	3	3	3	1
Lake McDermott	5.46	1	1	0	0	2	2	1	1	1	1	1	1	0	0	0	0	1	0	0	0
Baandee south	6.08	0	0	0	0	3	3	3	2	3	3	3	2	3	3	1	1	3	3	3	2
Baandee north	7.07	3	2	2	2	3	2	2	2	2	2	2	2	3	3	3	2	3	3	1	1
Lake De Courcy	7.66	1	0	0	0	3	3	3	3	1	2	1	0	1	1	2	1	3	3	3	1
North Lake Grace	7.98	1	0	0	0	1	0	0	0	0	0	0	0	0	0	0	0	3	0	0	0
Lake Lucille	7.99	0	0	0	0	0	0	1	0	2	1	1	1	0	0	0	0	1	0	0	0
Lime Lake	8.10	2	2	0	0	3	3	2	2	2	2	1	1	2	2	2	2	1	2	2	1
Kurrenkutten	8.18	0	0	0	0	1	1	1	1	1	1	0	0	1	1	0	0	1	0	0	0
Lake Grace	8.37	1	0	0	0	1	2	1	1	0	0	0	0	3	3	1	1	3	3	3	1
Jilakin	8.38	1	3	2	2	2	2	1	1	2	1	1	1	3	3	3	3	3	3	3	3
Dumbleyung	8.52	0	0	0	1	1	0	0	0	1	1	1	1	2	2	2	2	3	2	3	3
Parkeyerring	8.53	1	0	0	0	0	1	0	0	1	0	0	0	1	2	1	1	3	3	1	0
Salt Lake	8.65	2	1	1	1	2	2	2	2	2	2	3	2	2	1	0	0	1	1	0	0

*Activity was assessed via visual observation of pH indicator color change according to the following scale: 0—none (no shading); 1—minor (light blue); 2—moderate (mid blue); 3—extensive (dark blue).*

Acidophilic iron oxidation cultures from alkaline saline lakes (Cluster B, sediment pH > 8) outperformed those from acidic saline lakes (Cluster A, sediment pH ≤ 8; Table 3). This was unexpected; however, OTU-level diversity was higher in alkaline saline lakes than acidic saline lakes (Supplementary Figure 4), and hence alkaline saline lakes may host communities with broader environmental tolerances and functional capacities than acidic counterparts simply by virtue of this higher diversity. This suggests that OTU-level diversity of microbial communities from saline lakes is more important than their present-day environmental conditions in determining their ability to survive and oxidize ferrous iron in acidic enrichment culture media. Remarkably, the highest iron-oxidizing activity was observed in one of the saltiest lakes, i.e., Salt Lake. Analysis of microbial community composition at lower taxonomic ranks within Cluster B communities did not identify known acidophilic iron oxidizers at particularly high (> 0.05%) relative abundances. Future work will include DNA sequencing to identify the species driving iron oxidation under these highly acidic and saline conditions.

Capacity for sulfur oxidation was more widely and evenly distributed than iron oxidation across enrichment cultures from both acidic and alkaline saline lakes (Table 4). Most lakes showed capacity for sulfur oxidation at pH 3–10 and salinities up to 160 g L^–1^ NaCl (Table 4). Sulfur oxidation was limited at pH 2, with less than a third of enrichments capable of sulfur oxidation at this pH above 20 g L^–1^ NaCl (Table 4). All Cluster B (and most Cluster A) communities hosted an unclassified *Thiohalorhabdus* sp., a putative halotolerant (up to 230 g L^–1^ NaCl) sulfur oxidizer at circumneutral pH (Sorokin et al., 2008), and all Cluster B (but not Cluster A) communities hosted an unclassified *Halothiobacillus* sp., a putative halotolerant sulfur oxidizer at acidic (pH 3.5) to alkaline (pH 9) conditions (Kelly and Wood, 2000; Sievert et al., 2000; Whaley-Martin et al., 2019). These known halotolerant sulfur oxidizers are likely responsible for the strong sulfur oxidation responses observed under widely varying pH and salinity conditions imposed in the enrichment cultures.

In general, little evidence for biotic iron oxidation was observed in microbial communities from lakes with pH < 8, which was unexpected as these were expected to host acidophiles capable of iron oxidation at the media pH values of pH 1.5–2.5 used here, based on previous studies enriching acidophilic iron oxidizers from acidic saline lakes (e.g., Rea et al., 2015). Our observations of acidophilic iron oxidation in cultures from lake sediments with pH > 8 were also unexpected, and highlight the genetic and functional diversity of these alkaline lake sediments. Sulfur oxidation capacity was more evenly spread among lakes, with no clear influence of lake sediment pH. These results emphasize the importance of wide geographical sampling to maximize recovery of novel culturable microbial diversity and function; and that current OTU-level diversity may be a better indicator of functional capacity and tolerances than the current environmental conditions.

### Scale-Dependent Associations With Microbial Community Composition Emphasize Sampling From Remote, High Diversity Communities When Bioprospecting

The main variable correlated with microbial community composition was scale-dependent. pH was most strongly correlated with microbial community composition over smaller spatial scales (less than 200 km between lakes); whereas geographical distance was most strongly correlated over larger spatial scales (greater than 200 km between lakes), in accordance with the ecological theory of distance-decay. Capturing the full genetic and functional diversity of salt lake microbial communities globally therefore, requires sampling and characterization of communities present in salt lakes geographically distant from those previously sampled. This extends the work of Viver et al. (2015) in characterizing cultivable aerobic heterotrophs in eight solar salterns across multiple continents, which demonstrated the need for sampling and cultivation from non-redundant environments to capture a greater breadth of microbial functional capacity. Here, we show that sampling and cultivation from non-redundant environments should prioritize sampling from the most geographically distant examples of these non-redundant environments to maximize recovery of novel cultivable microbial diversity. This study contributes to extending known microbial diversity, functional capacity, and environmental tolerances in salt lakes by focussing on Western Australian salt lakes, poorly characterized to date in comparison with, and distant from, other major foci of salt lake microbiological research such as the East African Rift Valley (Rees et al., 2004) and Egypt (Mesbah et al., 2007), western United States of America (Ley et al., 2006; Harris et al., 2013; Tazi et al., 2014), China (Dong et al., 2006; Jiang et al., 2007; Pagaling et al., 2009; Xiong et al., 2012, 2014; Yang et al., 2016; Han et al., 2017), Spain (Casamayor et al., 2013; Montoya et al., 2013), southeastern Australia (Lake Tyrrell, Victoria; Narasingarao et al., 2012; Podell et al., 2014), and Russia (Sorokin et al., 2008; Vavourakis et al., 2016). Indeed, the substantially higher salinity tolerances of iron- and sulfur-oxidizing enrichment cultures from the Western Australian salt lakes compared with previous known tolerances demonstrates the value of characterizing remote locations. Surprisingly, culturable functions and tolerances were not well correlated with current environmental conditions at the lakes. Instead, OTU level diversity in the salt lake microbial communities emerged as the major indicator of iron- and sulfur-oxidizing capacity and environmental tolerances to extremes of pH and salinity. Overall, when bioprospecting for novel microbial functional capacity and environmental tolerances, our study supports sampling from remote, previously unexplored locations, and prioritizing for OTU level diversity rather than present geochemical conditions.

## Data Availability Statement

The datasets presented in this study can be found in online repositories. The names of the repository/repositories and accession number(s) can be found below: https://www.ncbi.nlm.nih.gov/, PRJNA563230.

## Author Contributions

TS, CZ, and GS secured the funding. TS and LG completed the fieldwork. LG, CZ, and TS analyzed the samples and data. All authors contributed to the writing of the manuscript and conceptualized the study.

## Conflict of Interest

The authors declare that the research was conducted in the absence of any commercial or financial relationships that could be construed as a potential conflict of interest.

## Publisher’s Note

All claims expressed in this article are solely those of the authors and do not necessarily represent those of their affiliated organizations, or those of the publisher, the editors and the reviewers. Any product that may be evaluated in this article, or claim that may be made by its manufacturer, is not guaranteed or endorsed by the publisher.
