# Supplementary material for: Microbial Community Structure Is Most Strongly Associated With Geographical Distance and pH in Salt Lake Sediments
Source: Front Microbiol. 2022 Jun 2;13:920056. doi: 10.3389/fmicb.2022.920056 (PMC9221066; doi:10.3389/fmicb.2022.920056)
Supplement: Supplementary file 2 [file Table_2.DOCX]

**Supplementary Material**

Title: Microbial community structure is mostly strongly associated with geographical distance and pH in salt lake sediments

Authors: Santini, TC, Gramenz, L, Southam, G, Zammit, C

Contents: 4 tables, 4 figures, 10 pages

**Supplementary Information Table 1.** Correlations among microbial community composition (‘microbial’), geographical distance (‘distance’), and geochemical variables (‘all geochem’: all geochemical variables; or ‘pH': pH only) as determined by Mantel and partial Mantel tests on dissimilarity matrices for subgroups of southern and northern sites (n=8 sites in each group). All tests were conducted with 9999 permutations. Significant correlations (p<0.05) are indicated with bold *P*-values.

|  | **Group 1: Southern**  **(SA2841-2848)** | | **Group 2: Northern**  **(SA2849-SA2857)** | |
| --- | --- | --- | --- | --- |
| **Comparisons** | **Mantel r** | ***P-*value** | **Mantel r** | ***P-*value** |
| *Mantel tests* |  |  |  |  |
| Distance x microbial | -0.1644 | 0.3305 | 0.0337 | 0.8601 |
| All geochem x microbial | 0.3492 | 0.2567 | 0.1165 | 0.6958 |
| pH x microbial | 0.4913 | **0.0266** | 0.2105 | 0.3633 |
| Distance x all geochem | 0.1664 | 0.3695 | 0.4015 | 0.0687 |
| Distance x pH | -0.0193 | 0.9322 | 0.4281 | **0.0098** |
| *Partial Mantel tests* |  |  |  |  |
| Distance x microbial, controlling for all geochem | -0.2408 | 0.9034 | -0.0144 | 0.5408 |
| Distance x microbial, controlling for pH | -0.1753 | 0.8485 | -0.0639 | 0.6844 |
| All geochem x microbial, controlling for distance | 0.3871 | 0.1496 | 0.1124 | 0.3362 |
| pH x microbial,  controlling for distance | 0.4645 | **0.0263** | 0.2171 | 0.1958 |


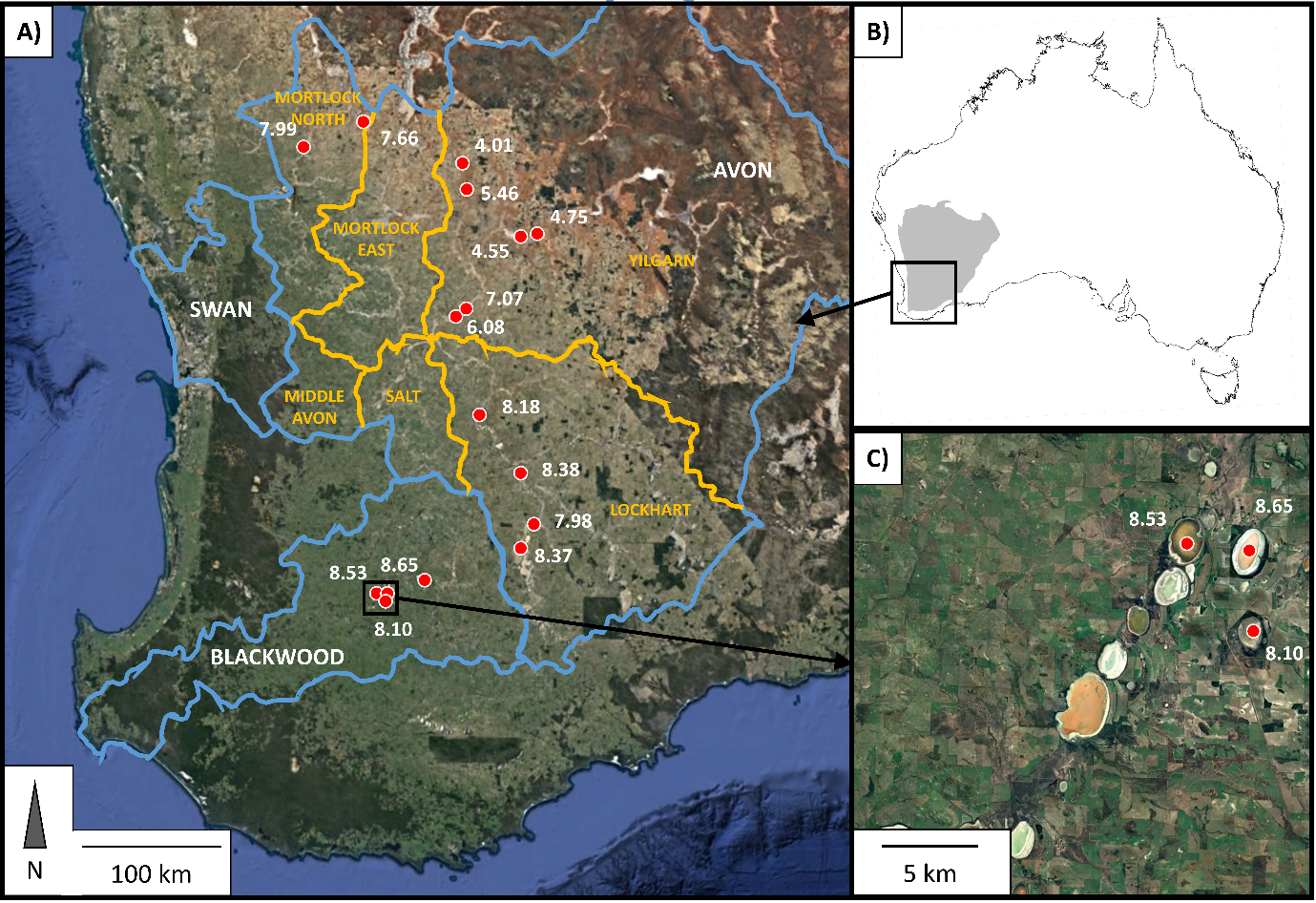


**Supplementary Information Figure 1.** Location of major drainage basins (boundaries indicated with blue lines and labelled in white capitalised text with catchment/basin names) and Avon sub-catchments (boundaries in yellow, labelled in yellow capitalised text with sub-catchment names) in relation to sampling sites (red dots) for this study. Source mapping from Degens and Shand (2010) and Hennig and Kelsey (2015).

**Supplementary Information Table 2.** Results of distance-based multivariate multiple regression (DistLM) based on Bray-Curtis dissimilarities for microbial community structures and measured geochemical variables, using 9999 permutations under a forward selection procedure with R^2^ as selection criterion. P-values and percentage of variation explained are for sequential tests. Geochemical variables are listed in order of selection. Percentages listed with dbRDA axes indicate the percentage of variation explained by each axis out of the fitted model and total variation.

| **Geochemical variables** | ***P*-value** | **Cumulative percentage of variation explained (%)** | **Multiple partial correlations with dbRDA axes** | |
| --- | --- | --- | --- | --- |
|  |  |  | **Axis 1**  **(34.6 % total)** | **Axis 2**  **(8.2 % total)** |
| pH | 0.006 | 17.99 | 0.116 | 0.291 |
| Water extractable Al | 0.092 | 26.38 | -0.023 | 0.054 |
| Total C (%wt) | 0.050 | 35.18 | 0.269 | -0.347 |
| Water extractable Fe | 0.186 | 41.69 | -0.026 | -0.112 |
| Water extractable K | 0.144 | 48.48 | 0.046 | 0.362 |
| Exchangeable Ca | 0.108 | 55.53 | 0.068 | 0.101 |
| Colwell K | 0.168 | 62.00 | -0.010 | -0.396 |
| Exchangeable Mg | 0.149 | 68.49 | 0.416 | 0.182 |
| Colwell P | 0.378 | 73.43 | 0.236 | -0.373 |
| Cation exchange capacity | 0.310 | 78.75 | 0.534 | -0.110 |
| Water extractable Cu | 0.342 | 83.68 | -0.003 | 0.011 |
| Water extractable Si | 0.401 | 88.14 | 0.130 | 0.266 |
| Sum of exchangeable cations | 0.408 | 92.46 | 0.053 | 0.091 |
| EC | 0.460 | 96.54 | -0.178 | 0.003 |

**Supplementary Information Table 3.** Results of distance-based multivariate multiple regression (DistLM) based on Bray-Curtis dissimilarities for microbial community structures and measured geochemical variables, using 9999 permutations under a forward selection procedure with AIC as selection criterion. P-values and percentage of variation explained are for marginal tests, with only those geochemical variables having a significant P value (p<0.05) listed here. Percentages listed with dbRDA axes indicate the percentage of variation explained by each axis out of the fitted model and total variation.

| **Environmental characteristic** | ***P*-value** | **Percentage of variation explained (%)** | **Multiple partial correlations with dbRDA axes** | |
| --- | --- | --- | --- | --- |
|  |  |  | **Axis 1**  **(34.6 % total)** | **Axis 2**  **(8.2 % total)** |
| pH | 0.0061 | 17.98 | 0.116 | 0.291 |
| CEC | 0.0242 | 14.29 | 0.534 | -0.110 |
| Water extractable Al | 0.0156 | 16.05 | -0.023 | 0.054 |


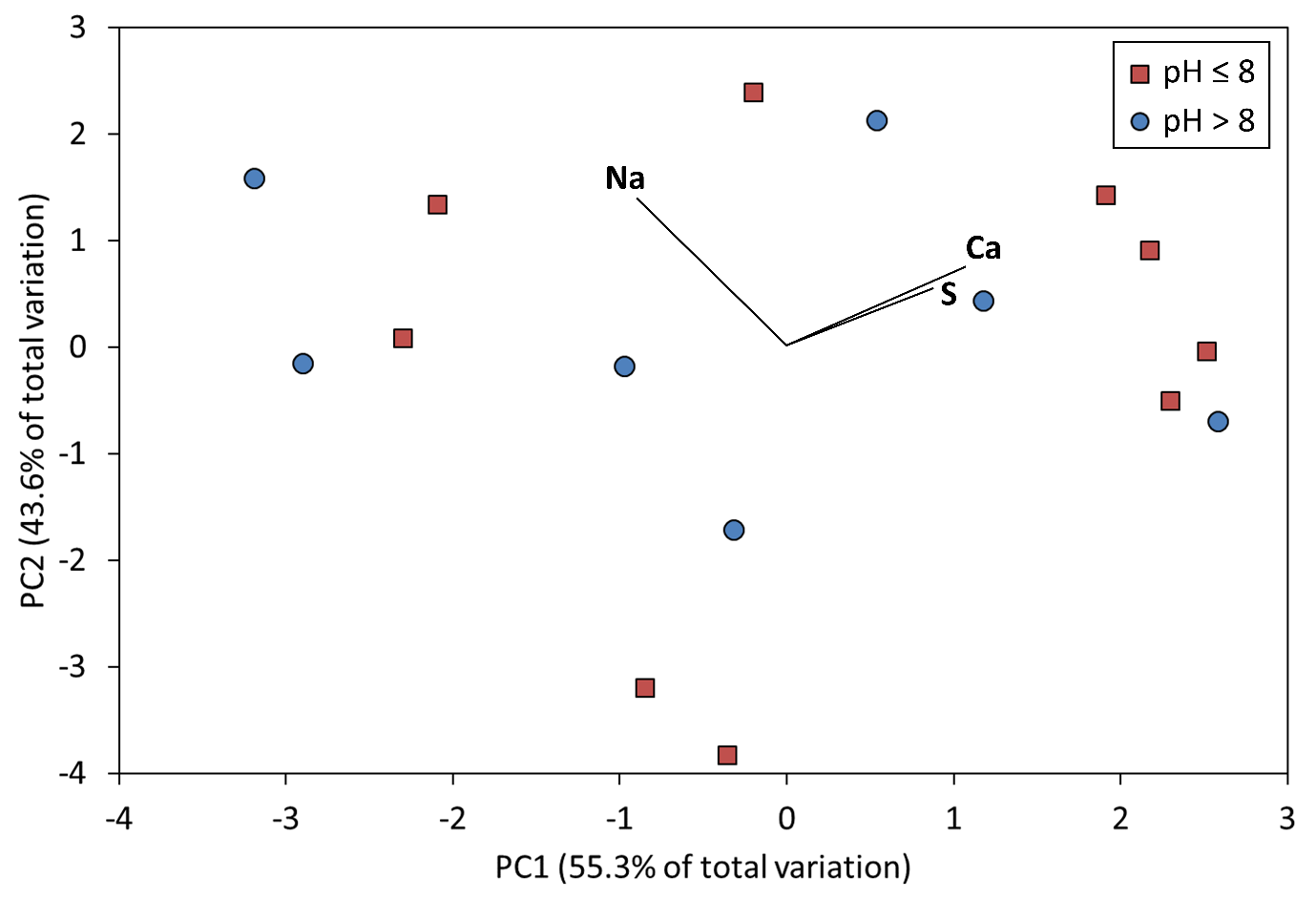


**Supplementary Information Figure 2.** Principal components plot (as visualised by principal component scores for each site) representing similarity in measured geochemical variables between Western Australian salt lakes. Each point shows score on principal components 1 and 2 for one lake. Lakes are split by sediment pH consistent with Figure 3: sites with pH 8 or below (marked with red squares, and sites with pH above 8 (marked with blue circles). Vectors for geochemical variables (in bold) with major contributions to PCs 1 and 2 are shown here.

**Supplementary Information Table 4.** Geographical locations and geochemical properties of sediment samples from all lakes sampled in this study. See attached Excel File.

**Supplementary Information Table 5.** Relative abundance of dominant OTUs in Clusters A and B of lake microbial communities as identified by PERMANOVA, and contributions to similarity within, and dissimilarity between, clusters as identified by SIMPER. SIMPER results for the ten OTUs making the greatest contribution to within-cluster similarity are displayed; those ten making the greatest contribution to Cluster A are shaded in grey; those contributing to Cluster B are unshaded. OTU31 was in the top ten in both Clusters A and B, and is shaded in grey. Dominant OTUs were defined as the five OTUs in each cluster with the highest relative abundance, and are highlighted in bold in the table below. ‘Rel Abund’ indicates the means of relative abundance (as a percentage of total sequence reads) across all sites within each cluster. ‘% Sim’ indicates the percentage contribution of each OTU to the overall similarity among microbial communities across all sites within each cluster. OTU contributions to between-cluster dissimilarity (‘% Dissim’) are listed in the final column, with the five most significant contributors marked in bold. OTU classifications are listed by phylum and then the lowest taxonomic rank assigned.

| **OTU** | **Cluster A: pH ≤ 8** | | **Cluster B: pH >8** | | **Cluster A and B dissimilarity** |
| --- | --- | --- | --- | --- | --- |
|  | **Rel Abund** | **% Sim** | **Rel Abund** | **% Sim** | **% Dissim** |
| *Bacillota*; *Lactobacillus* sp. 1 | **10.37** | **16.15** | 0.07 | 0.03 | **6.37** |
| *Bacillota*; *Clostridiaceae* sp. | **3.79** | **6.83** | 0.10 | 0.03 | 2.28 |
| *Pseudomonadota*; *Enterobacteriaceae* sp. | **3.29** | **5.96** | 0.03 | 0.01 | 2.02 |
| *Actinomycetota*; *Propionibacteriaceae* sp. | **3.51** | **5.71** | 0.01 | 0.001 | 2.16 |
| *Bacillota*; *Lactobacillus* sp. 2 | 2.66 | 4.70 | 0.03 | 0.01 | 1.62 |
| *Bacteroidota*; *Bacteroides* sp. | 2.57 | 4.54 | 0.01 | 0.001 | 1.59 |
| *Bacillota*; *Lachnospiraceae* sp. 1 | **2.68** | **4.14** | 0.06 | 0.01 | 1.62 |
| *Euryarchaeota*; *Halobacteriaceae* sp. 1 | 2.49 | 2.69 | **12.49** | **14.85** | **6.20** |
| *Bacteroidota*; *Bacteroidales* sp. | 1.86 | 2.24 | 0.01 | 0.001 | 1.15 |
| *Bacillota*; *Lachnospiraceae* sp. 2 | 1.23 | 1.78 | 0.02 | 0.01 | 0.75 |
| *Bacteroidota*; *Salinibacter* sp. | 1.09 | 0.98 | **7.25** | **7.40** | **3.84** |
| *Gemmatimonadota*; *Gemmatimonadota* sp 1. | 1.14 | 0.43 | **6.35** | **5.77** | **3.53** |
| *Euryarchaeota*; *Natronomonas* sp. | 1.24 | 1.70 | **4.49** | **5.58** | 2.03 |
| *Euryarchaeota*; *Halorhabdus* sp. | 1.15 | 1.04 | **4.87** | **5.36** | 2.39 |
| *Pseudomonadota*; *Rhodovibrio* sp. | 1.19 | 0.71 | 2.73 | 3.02 | 1.41 |
| *Euryarchaeota*; *Halobacteriaceae* sp. 2 | 0.48 | 0.55 | 2.12 | 2.81 | 1.02 |
| *OP1*; *OP1* sp. | 4.97 | 0.42 | 2.69 | 2.76 | **3.98** |
| *Pseudomonadota*; *Desulfohalobiaceae* sp. | 0.55 | 0.47 | 2.02 | 2.50 | 0.96 |
| *Balneolaeota*; *Balneolaceae* sp. | 0.33 | 0.39 | 1.86 | 2.22 | 0.95 |


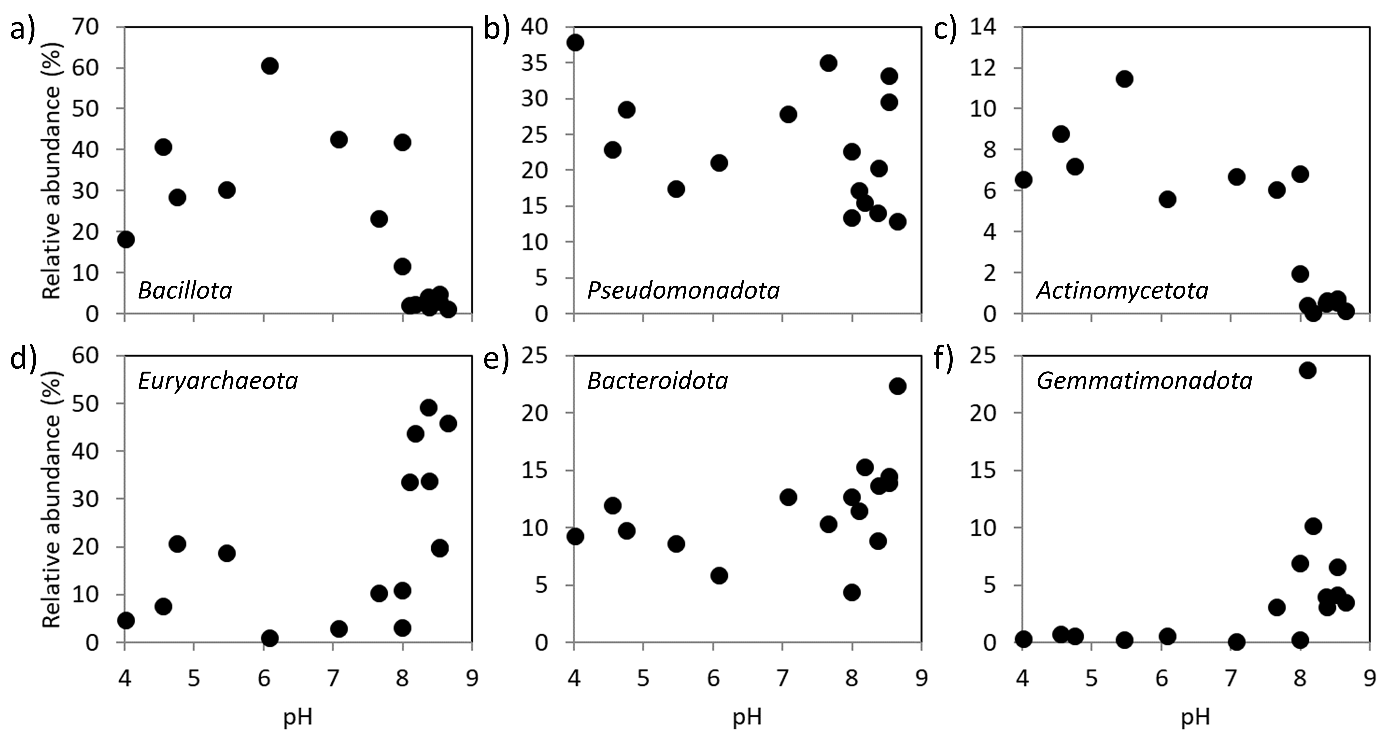


**Supplementary Information Figure 3.** Relative abundance of dominant phyla within Cluster A (sediment pH ≤ 8; panels a-c: *Bacillota*, *Pseudomonadota*, *Actinomycetota*) and Cluster B (sediment pH > 8; panels d-f: *Euryarchaeota*, *Bacteroidota*, *Gemmatimonadota*), as determined by PERMANOVA and SIMPER.


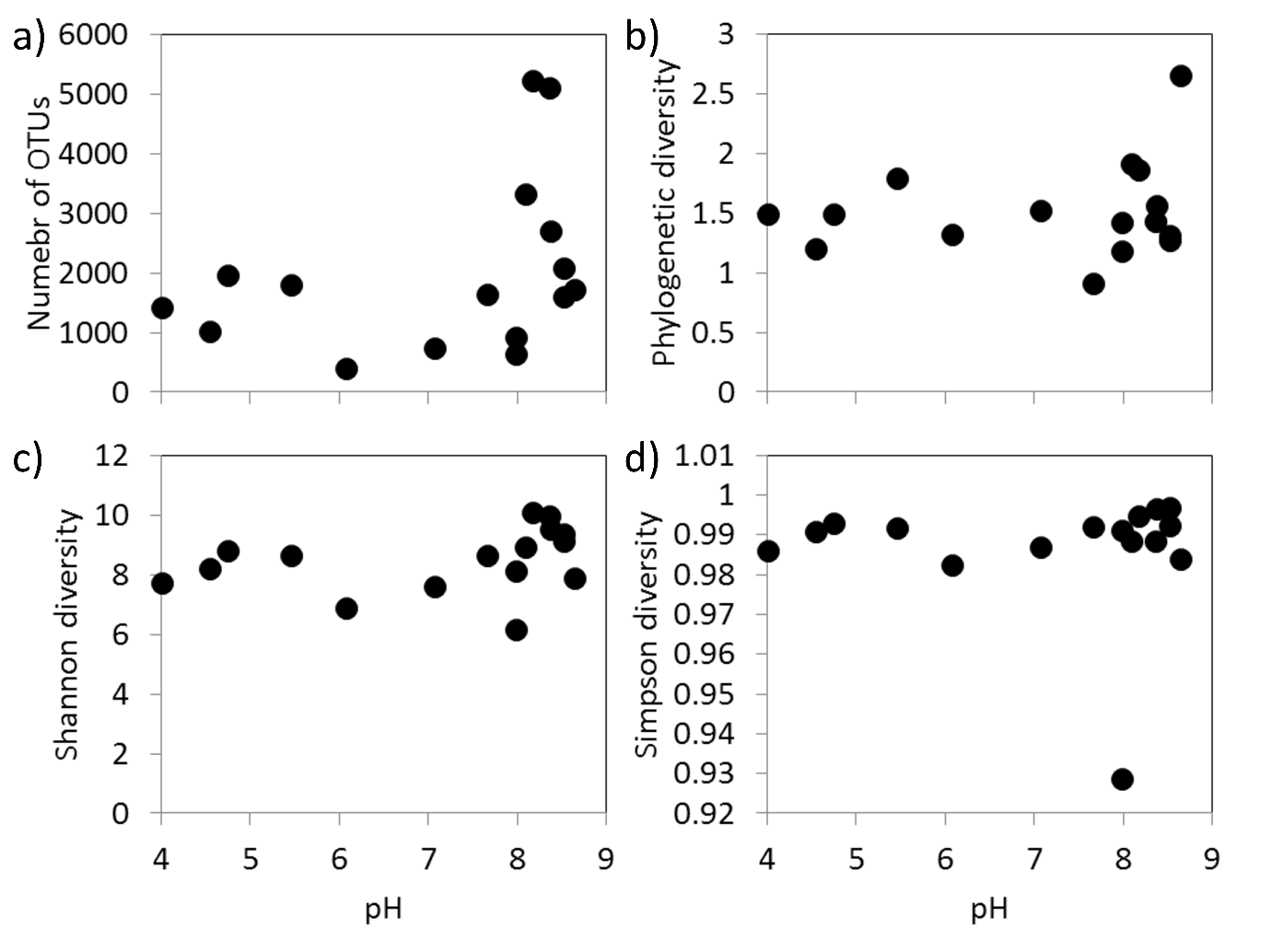


**Supplementary Information Figure 4.** Diversity metrics for salt lake microbial communities as a function of sediment pH: (a) numbers of OTUs (clustered at 97% sequence similarity); (b) Faith’s Phylogenetic Diversity; (c) Shannon diversity; and (d) Simpson diversity.

**References**

Hennig, K., Kelsey, P. (2015). Avon Basin hydrological and nutrient modelling. Water Science Technical Series, report no. 74. Perth: Department of Water.

Degens, B., Shand, P. (2010). Assessment of acidic saline groundwater hazard in the Western Australian Wheatbelt: Yarra Yarra, Blackwood, and South Coast. Floreat: CSIRO.
